# Pathological features of the placenta in hypothyroidism during pregnancy: a population-based retrospective cohort study in Chinese population

**DOI:** 10.3389/fendo.2025.1671641

**Published:** 2025-11-06

**Authors:** Zhuo Bao, Yingbin Li, Tong Xue, Yixuan Liu, Jinxiang Yan, Xiaoqian Wang, Yunling Li, Xianxu Zeng, Chengquan Zhao, Yongzhen Guo, Dongmei Lei

**Affiliations:** 1 Department of Pathology, The Third Affiliated Hospital of Zhengzhou University, Zhengzhou, China; 2 Zhengzhou Key Laboratory of Gynecological Disease’s Early Diagnosis, Zhengzhou, China; 3 Department of Pathology, Magee-Womens Hospital, University of Pittsburgh Medical Center, Pittsburgh, PA, United States; 4 Henan Province Engineering Research Center of Precision Diagnosis and Artificial Intelligence of Gynecological Tumors, Zhengzhou, Henan, China

**Keywords:** maternal hypothyroidism, placenta pathology, placental morphology, fetal vascular malperfusion, maternal vascular malperfusion

## Abstract

**Background:**

Pregnant women, due to changes in their metabolic and immune states, are a high - risk group for thyroid diseases. The most common one among them is hypothyroidism. However, there are few reports on its impact on placental morphology. This study aims to assess the effect of hypothyroidism on the gross morphology and histopathology of the placenta compared with gestational age - matched controls.

**Methods:**

Placental samples from women with singleton pregnancies who gave birth at the Third Affiliated Hospital of Zhengzhou University from June 2022 to December 2023 were collected. A total of 852 participants were recruited according to the inclusion and exclusion criteria, with 213 in the hypothyroidism group and 639 in the control group. Baseline demographic and clinical data of the pregnant women, as well as neonate-related information, were recorded. Gross placental measurements and histological sections from standardized placental samples were evaluated and statistically analyzed.

**Results:**

Overall, compared with the control group, insufficient vascular perfusion and inflammation were more frequently observed in the placentae of pregnant women with hypothyroidism. These included retroplacental hemorrhage (4.69% versus 1.25%, *P* = 0.002) related to maternal vascular malperfusion, delayed villous maturation (30.52% versus 17.37%, *P*<0.001) and decreased vasculosyncytial membrane (32.39% versus 24.10%, *P* = 0.017) related to fetal vascular malperfusion, chronic chorioamnionitis (7.04% versus 1.56%, *P*<0.001), and villitis of unknown etiology (3.29% versus 0.94%, *P* = 0.016). Additionally, hypothyroidism increased the risk of fetal complications, including fetal distress (15.49% versus 9.68%, *P* = 0.024) and small for gestational age (8.45% versus 4.54%, *P* = 0.030).

**Conclusion:**

Gestational hypothyroidism can lead to histopathological abnormalities in the placenta. These abnormalities affect the development and function of the placenta, consequently influencing perinatal outcomes and fetal development.

## Introduction

Hypothyroidism is one of the most common endocrine disorders, and is defined as a systemic metabolic syndrome resulting from a reduction in the synthesis or secretion of thyroid hormones for a variety of etiologies (1, 2). It affects approximately 3-5% of pregnant women, with its prevalence being even higher in iodine-deficient areas due to pregnancy-induced alterations in hormone levels and iodine metabolism which can disrupt maternal thyroid function (3–5). Thyroid hormones participate in and regulate various metabolic activities, playing an important role in pregnant women. Adequate thyroid hormone is crucial for fetal brain development. In early pregnancy, the thyroid hormones involved in fetal growth and development are entirely of maternal origin (4). Therefore, hypothyroidism during pregnancy has consistently been shown to be associated with adverse obstetric and neonatal outcomes, including maternal anemia, pre-eclampsia, premature birth, low birth weight, placental abruption, increased rates of NICU admission, respiratory distress syndrome, and impaired fetal neurocognitive development (6–8).

The placenta is a unique and complex organ that serves as a temporary support organ connecting the mother and fetus during pregnancy, performing the nutrient-supplying and endocrine function (9). Various diseases, including infections, metabolic, genetic, circulatory, and maturation defects, may affect its function (10). Adequate placental function is crucial for good pregnancy outcomes and optimal fetal development. Deficiencies in placental function can result in a variety of common pregnancy complications, which can often be detected through a comprehensive pathological examination of the placenta (11). Placental pathology can be generally divided into vascular and inflammatory lesions. Vascular lesions include maternal and fetal subgroups, each of which may manifest as malperfusion-related lesions, disruption of vascular integrity or resulting damage to placental parenchyma. In addition, acute and chronic inflammatory lesions can be subdivided into infectious and idiopathic (11, 12).

Alterations in placental growth and development are also influenced by maternal thyroid hormone levels (13). Thus, hypothyroidism in pregnancy affects placental function, which in turn leads to fetal growth restriction. While the association between hypothyroidism and adverse pregnancy outcomes is well-established, systematic studies characterizing its specific impacts on placental morphology remain limited. Recent studies have begun to reveal its pathological basis from different perspectives. Hypothyroidism has been associated with bilobed placenta, retroplacental haematoma, decidual arteriopathy, and inflammatory lesions in specific populations (such as *in vitro* fertilization pregnancies) (14). Meanwhile, studies on thyroid autoimmunity have shown that pathological features such as abnormal uterine artery blood flow, maternal vascular malperfusion, inflammatory response, and oxidative stress may occur in the placenta (15, 16). At a mechanistic level, gestational hypothyroidism has been associated with the increased trophoblast apoptosis, delayed villus maturation and abnormal angiogenesis (17). However, for the general obstetric population, there remains a lack of comprehensive and systematic descriptions of the characteristic pathological lesions caused by maternal hypothyroidism during pregnancy. In this study, we analyzed and compared the placental morphological characteristics of pregnant women with hypothyroidism and those with healthy pregnancies to determine the effect of hypothyroidism during pregnancy on placental morphology.

## Materials and methods

### Study design and patient selection

This was a retrospective cohort study including the pregnant women with hypothyroidism (diagnosed gestationally and managed with levothyroxine (LT4) to sustain euthyroidism), who delivered at the Third Affiliated Hospital of Zhengzhou University from June 2022 to December 2023. Pregnant women with no hypothyroidism during the same period were included as the control group. The subjects in the control group were matched with the hypothyroidism group in a ratio of 3:1 according to the gestational age at delivery. The following conditions were excluded from both study and control groups: (1) having other complications such as diabetes, hypertension, hyperthyroidism, Hashimoto’s thyroiditis, autoimmune diseases, cardiovascular diseases, respiratory system diseases, and chronic kidney diseases; (2) having amniotic fluid contamination, severe intrahepatic cholestasis of pregnancy; (3) twin or multiple births; (4) infections such as hepatitis B, hepatitis C, syphilis, and Human Immunodeficiency Virus (HIV) infection.

Based on the selection criteria, 852 pregnant women were selected in this study, including 213 cases in the hypothyroidism group and 639 cases in the control group. Clinical data, such as maternal age, gravidity, parity, mode of delivery, and perinatal outcomes, were collected and presented as baseline characteristics in **Table 1**. The study was granted ethical approval by the Ethics Committee of the Third Affiliated Hospital of Zhengzhou University (2022-111-01, 2022-258-01).

**Table 1:** Maternal and fetal characteristics of hypothyroidism group and control group.

Characteristics	Hypothyroidism Cases n = 213	Controls n = 639	P value
Maternal age (years, Median [IQR])	32.00 (29.00 - 34.00)	32.00 (29.00 - 34.00)	0.404
Maternal BMI (kg/m^2^, Median [IQR])	26.64 (24.82 - 29.30)	26.85 (24.84 - 29.30)	0.705
Maternal gain weight (kg, Median [IQR])	14.00 (11.00 - 17.00)	14.00 (10.00 - 16.00)	0.097
Gravidity (n, %)			0.015
≤2	151 (70.89)	394 (61.66)	
>2	62 (29.11)	245 (38.34)	
Parity (n, %)			0.052
≤2	201 (94.37)	575 (89.98)	
>2	12 (5.63)	64 (10.02)	
Delivery mode (n, %)			0.406
Vaginal Delivery	66 (30.99)	179 (28.01)	
Caesarean Section	147 (69.01)	460 (71.99)	
Neonatal gender (n, %)			0.089
Boy	117 (54.93)	308 (48.20)	
Girl	96 (45.07)	331 (51.80)	
Neonatal weight (g, Median [IQR])	3280.00 (2992.50 - 3567.50)	3265.00 (3030.00 - 3575.00)	0.759
Neonatal length (cm, Median [IQR])	51.00 (50.00 - 52.00)	51.00 (50.00 - 52.00)	0.376
1-min Apgar score (n, %)			0.684
>7	212 (99.53)	636 (99.53)	
≤7	1 (0.47)	3 (0.47)	
5-min Apgar score (n, %)			0.750
>7	213 (100)	638 (99.84)	
≤7	0 (0.00)	1 (0.16)	
Fetal distress (n, %)	33 (15.49)	63 (9.86)	0.024
FGR (n, %)	4 (1.88)	10 (1.56)	0.756
SGA (n, %)	18 (8.45)	29 (4.54)	0.030
LGA (n, %)	33 (15.49)	90 (14.08)	0.613

IQR, interquartile range; BMI, Body Mass Index; FGR, fetal growth restriction; SGA, small for gestational age; LGA, large for gestational age; SD, standard deviation.

### Placental gross and histopathological examinations

Gross examination was performed for all placentae, including the gross morphological description and placental measurements, as well as the placental weight before and after trimming of the chorioamniotic membranes and umbilical cord. The collected placentae underwent standardized sampling according to the consensus statement of the Amsterdam Placenta Symposium group (18). Hematoxylin and eosin (H&E) stained slides were reviewed by two experienced pathologists for a consensus using the Olympus BX53 light microscope.

All placental lesions were pathologically diagnosed according to the standardized classification and diagnostic criteria established by the Amsterdam Consensus.

### Statistical analysis

Data were analyzed using Statistical Package for the Social Sciences (SPSS) software version 26.0 (PASW Statistics, USA). The normality of all continuous variables was assessed using the Shapiro-Wilk test. Since the data significantly deviated from a normal distribution (*P* <.05 for most variables), continuous data are presented as median and interquartile range (IQR). Differences between the hypothyroidism and control groups were compared using the Mann-Whitney U test for continuous variables. Categorical data are presented as number (percentage) and were compared using the chi-square test or Fisher’s exact test. To evaluate the association between hypothyroidism and the risk of key outcomes (e.g., placental lesions), binary logistic regression was used to calculate odds ratios (ORs) and 95% confidence intervals (95% CIs). A *P* value < 0.05 was considered statistically significant.

## Results

### Baseline maternal and fetal characteristics of participants

**Table 1** presents a comparative analysis of maternal and neonatal baseline characteristics for subjects within both the hypothyroidism and the control groups. Of the 852 participants, the mean maternal age was comparable between the hypothyroidism and control groups, while the gravidity in the hypothyroidism group was slightly lower than that observed in the control group. Other maternal characteristics, including body mass index (BMI), gestational weight gain, parity, and mode of delivery were similarly distributed between hypothyroidism and control groups. In terms of neonatal clinical characteristics, the hypothyroidism group exhibited significantly higher incidences of fetal distress (15.49% vs 9.68%, *P* = 0.024) and small for gestational age (SGA) (8.45% vs 4.54%, P = 0.030) compared to the control group, with no statistical differences in other indicators.

### Distribution of placental gross morphological indicators

The placental gross morphological characteristics are present in **Table 2**. The gross characteristics of the placenta, such as placental weight, placental efficiency (fetal-placental weight ratio), placental thickness, umbilical cord diameter, and other related factors, showed no statistical difference between the hypothyroidism group and the control group.

**Table 2:** Placental gross morphological indicators in hypothyroidism group and control group.

Parameters	Hypothyroidism cases n = 213	Controls n = 639	P value
Placental weight (g, Median [IQR])	544.80 (472.20 - 622.00)	543.70 (476.20 - 620.70)	0.885
Placental efficiency (Median [IQR])	5.94 (5.39 - 6.61)	6.01 (5.39 - 6.67)	0.391
Placental length (cm, Median [IQR])	20.00 (18.25 - 21.00)	20.00 (18.50 - 21.50)	0.501
Placental thickness (cm, Median [IQR])	2.00 (2.00 - 2.50)	2.20 (2.00 - 2.50)	0.806
Cord diameter (cm, Median [IQR])	1.20 (1.00 - 1.50)	1.20 (1.00 - 1.50)	0.650
Spiral diameter (ratio, Median [IQR])	0.15 (0.09 - 0.23)	0.16 (0.10 - 0.22)	0.818
Rotation direction (n, %)			0.144
Left	167 (81.07)	530 (85.35)	
Right	91 (14.65)	39 (18.93)	
Number of blood vessels (n, %)			0.687
2	1 (0.47)	6 (0.94)	
3	212 (99.53)	633 (99.06)	

IQR, interquartile range.

### Placental histopathological characteristics

**Figure 1:**
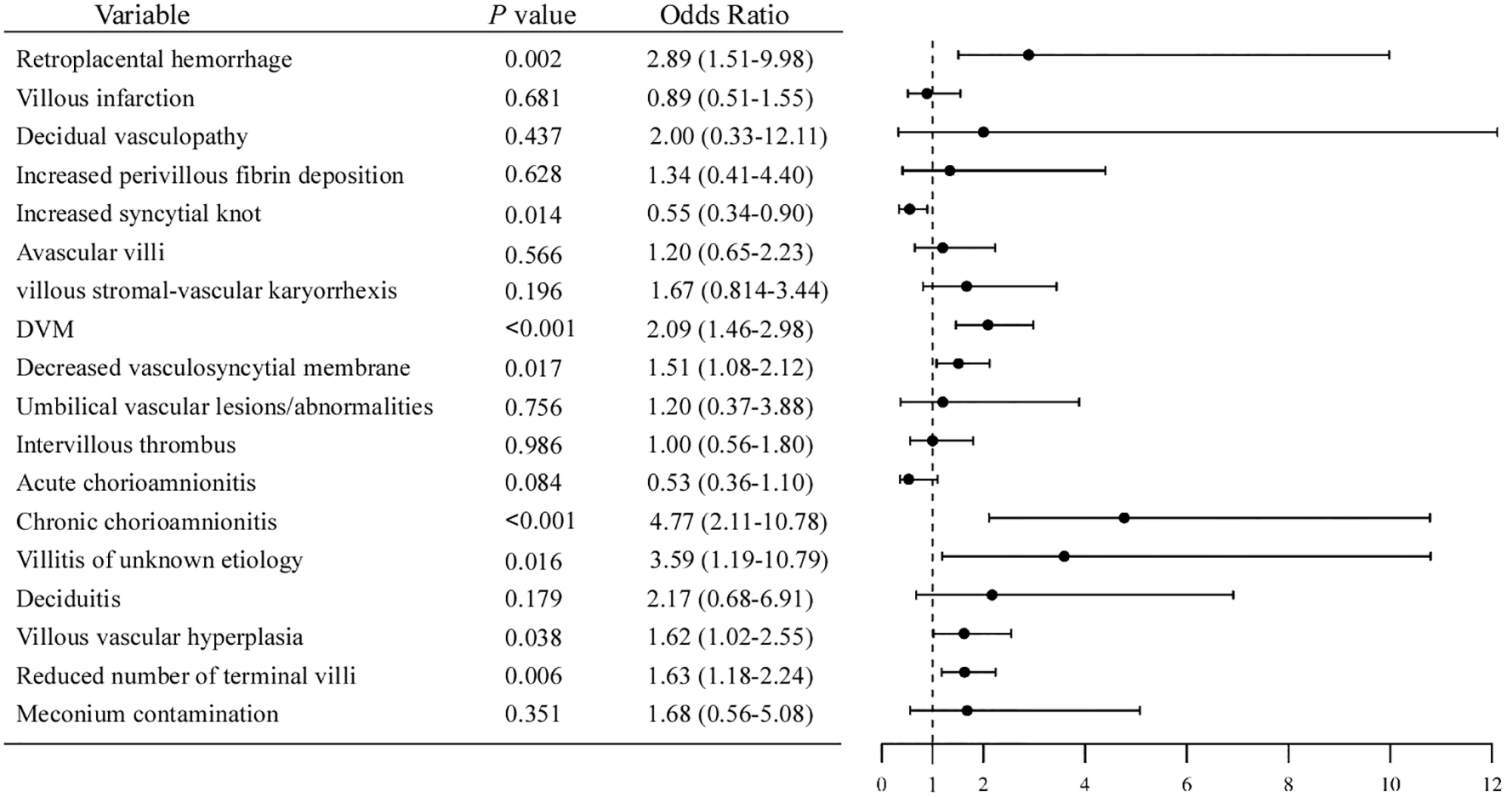
Forest plot of odds ratios for placental pathological features associated with hypothyroidism.

Microscopically, placental histological findings were categorized into maternal vascular malperfusion (MVM), fetal vascular malperfusion (FVM), inflammatory lesions, and other subgroups based on the Amsterdam Placental Workshop Group Consensus. The histopathologic placental findings were displayed in **Table 3**. Odds ratios (ORs) and 95% confidence intervals (CIs) of the associations between hypothyroidism and various placental pathological features were estimated, and the results are summarized in **Figure 1**.

**Table 3:** Placental histomorphological characteristics in hypothyroidism placentae compared with the control group.

Parameters	Hypothyroidism cases n = 213	Controls n = 639	P value
MVM			
Retroplacental hemorrhage	10 (4.69%)	8 (1.25%)	0.002
Villous infarction	18 (8.45%)	60 (9.39%)	0.681
Decidual vasculopathy	2 (0.94%)	3 (0.47%)	0.437
Increased perivillous fibrin deposition	4 (1.88%)	9 (1.41%)	0.628
Increased syncytial knot	22 (10.33%)	111 (17.37%)	0.014
FVM			
Avascular villi	15 (7.04%)	38 (5.95%)	0.566
villous stromal-vascular karyorrhexis	12 (5.63%)	22 (3.44%)	0.196
DVM	65 (30.52%)	111 (17.37)	<0.001
Decreased vasculosyncytial membrane	69 (32.39%)	154 (24.10%)	0.017
Umbilical vascular lesions/abnormalities	4 (1.88%)	10 (1.56%)	0.756
Intervillous thrombus	16 (7.51%)	48 (7.51%)	0.986
Inflammation			
Acute chorioamnionitis	9 (4.23%)	49 (7.67%)	0.084
Chronic chorioamnionitis	15 (7.04%)	10 (1.56%)	<0.001
Villitis of unknown etiology	7 (3.29%)	6 (0.94%)	0.016
Deciduitis	5 (2.35%)	7 (1.10%)	0.179
Others			
Villous vascular hyperplasia	32 (15.02%)	63 (9.86%)	0.038
Number of terminal villi			0.006
20-30	91 (42.72%)	201 (31.46%)	
40-60	122 (57.28%)	438 (68.54%)	
Meconium contamination	5 (2.35%)	9 (1.41%)	0.351

MVM, maternal vascular malperfusion; FVM, fetal vascular malperfusion; DVM, delayed villous maturation.

Among the MVM lesions, the incidence of retroplacental hemorrhage in pregnant women with hypothyroidism was 4.69%, significantly higher than the 1.25% in the control group (OR = 2.89, 95% CI [1.51, 9.98], *P* = 0.002). While the incidence of increased syncytial knots was 10.33% in the hypothyroidism group, which was notably less than the 17.37% in the control group (OR = 0.55, 95% CI [0.24, 0.90], *P* = 0.014). In FVM lesions, compared with the control group, the hypothyroidism group had significantly higher incidences of delayed villous maturation (DVM) (30.52% vs 24.10%; OR = 2.09, 95% CI [1.46, 2.98], *P* < 0.001) and reduced vasculosyncytial membrane (32.39% vs 24.10%; OR = 1.51, 95% CI [1.08, 2.12], *P* = 0.017).

For placental inflammation, the incidence of chronic chorioamnionitis (CCA) and villitis of unknown etiology (VUE) in hypothyroidism group were 7.04% (15/213) and 3.29% (7/213), respectively, which were significantly higher than that in the control group (CCA: OR = 4.77, 95% CI [2.11-10.78], *P* < 0.001; VUE: OR = 3.59, 95%CI [1.19-10.79], *P* = 0.016). However, the incidence of acute chorioamnionitis was marginally higher in the control group than in the hypothyroidism group, which was not statistically significant.

Compared to the control group, the hypothyroidism group exhibited a higher incidence of placental villous vascular hyperplasia (OR = 1.62, 95% CI [1.02, 2.55], *P* = 0.038) and a reduced number of terminal villi (OR = 1.63, 95% CI [1.18, 2.24], *P* = 0.003).

**Figure 2:**
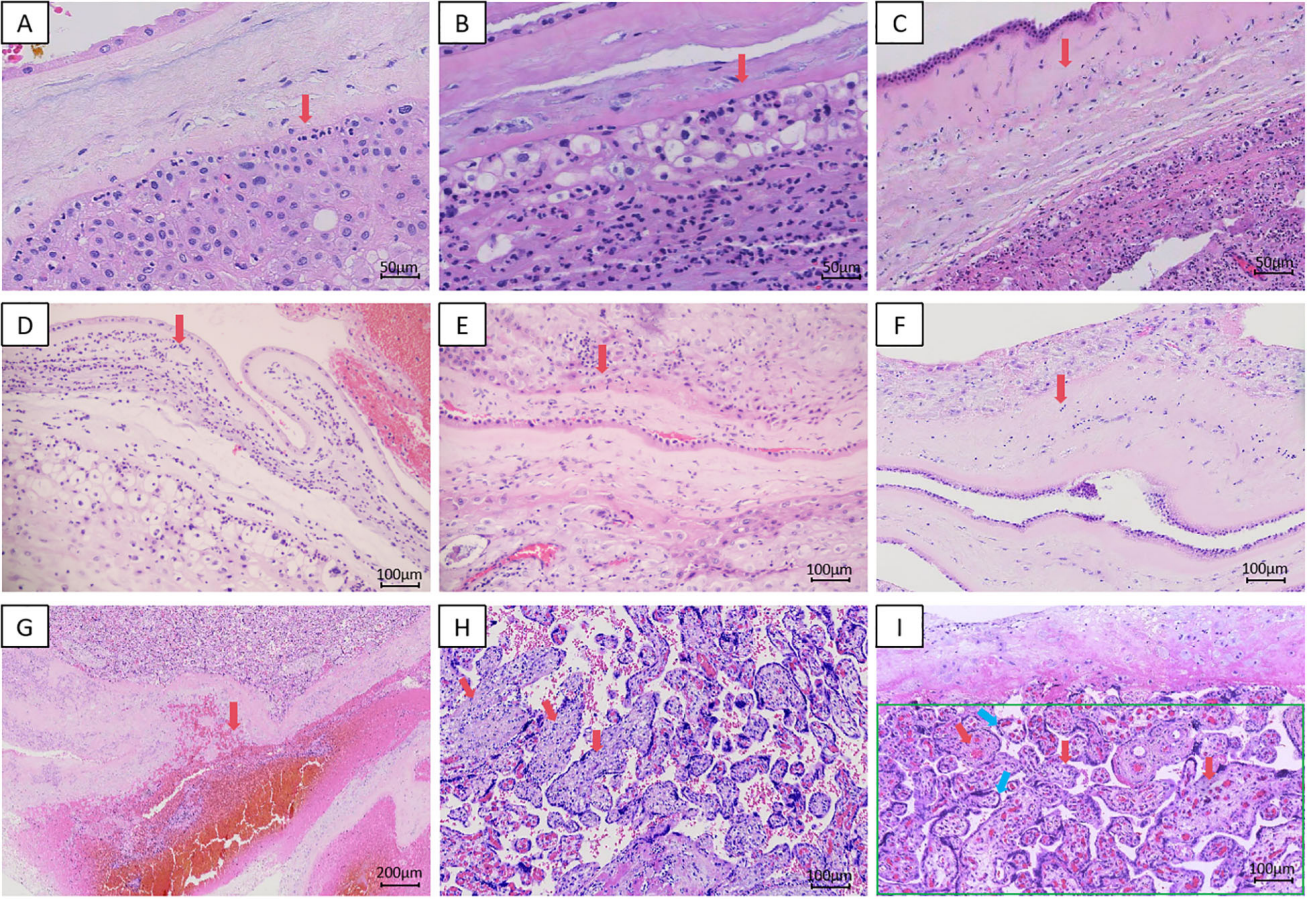
Representative HE-stained histopathological sections of placental tissue. **(A)** Acute chorioamnionitis,Stage 1, Grade 1, 200x; **(B)** Acute chorioamnionitis,Stage 1, Grade 2, 200x; **(C)** Acute chorioamnionitis,Stage 2, Grade 1, 200x; **(D)** Acute chorioamnionitis,Stage 2, Grade 2, 100x; **(E)** Chronic chorioamnionitis,Stage 1, Grade 1,100x; **(F)** Chronic chorioamnionitis,Stage 2, Grade 1,100x; **(G)** Retroplacental Hematoma, 40x; **(H)** VUE, 100x; **(I)** Delayed Villous Maturation (red arrow); Decreased Formation of Vasculosyncytial Membranes (blue arrow); Decreased Formation of Terminal Villi (green box), 100x.

The representative histopathological images with H&E staining are presented in **Figure 2**.

## Discussion

This matched cohort study of pregnant Chinese women provided an assessment of the associations between hypothyroidism during pregnancy and the gross morphological and histopathological changes of the placenta. We found that compared with the control group, women with hypothyroidism during pregnancy had a higher risk of retroplacental hemorrhage, DVM, decreased vasculosyncytial membrane, CCA, VUE, and villous vascular hyperplasia.

Hypothyroidism may increase the risk of infertility in women of childbearing age (19). As TSH levels rise in early pregnancy, the risk of miscarriage gradually increases (4). The baseline data of pregnant women in the hypothyroidism and the control group in this study showed that pregnant women with hypothyroidism had fewer gravidity than normal pregnant women, which may be relevant. Furthermore, data also showed that the incidence of fetal distress and SGA infants was higher in the gestational hypothyroidism group. Thyroid hormone is critical for fetal growth and development. During pregnancy, hypothyroidism can cause fetal growth retardation and neurodevelopmental abnormalities; it may also induce placental dysfunction, impair fetal blood supply, and lead to intrauterine hypoxia and subsequent fetal distress (3, 19, 20).

This study found that in the placenta of hypothyroidism group, retroplacental hemorrhage related to MVM, as well as delayed villous maturation and reduced vasculosyncytial membrane related to FVM were more frequently observed. These might represent manifestations of impaired placental vascularization. Thyroid hormones have the effect of promoting angiogenesis, and thyroid function in early to mid-pregnancy is associated with placental vascular development (21). It has been reported in research that maternal hypothyroidism can affect the expression of placental angiogenic factors and hormonal factors produced by the placenta, such as vascular endothelial growth factor (VEGF), placental growth factor (PlGF), placental lactogen-1 (PL-1), resulting in the inhibition of placental angiogenesis and the process of fetus-placenta development (22). Likewise, animal studies on hypothyroidism rats during pregnancy have shown reduced proliferation of cytotrophoblast cells and invasion of extravillous trophoblast cells, as well as decreased expression of cytokines, matrix metalloproteinases, nitric oxide synthase, and leptin. These molecules and enzymes are critical for remodeling the uterine spiral arteries and supporting placental development (23–25). Notably, the failure of spiral artery remodeling is the key event of MVM, which can lead to increased blood flow resistance and vascular structural damage (26), this elevates the risk of retroplacental hemorrhage. Furthermore, the reduction in placental angiogenesis impairs the formation of vasculosyncytial membranes (the critical diffusion barrier between the maternal and fetal circulations). Decreased vasculosyncytial membrane counts can affect the diffusion of oxygen and nutrients, thereby increasing the possibility of fetal hypoxia, which in turn induces delayed villous maturation (27).

In addition, our study also observed that the incidence of villous vascular hyperplasia was higher in the hypothyroidism group, while a reduction in terminal villi, which are the final nodes of the fetal circulation. This further reflects insufficient placental perfusion. Thyroid hormone deficiency during hypothyroidism compromises placental vascularization and trophoblast differentiation, which disrupts the normal maturation of intermediate villi into terminal villi. The consequent reduction in terminal villi, consistent with the observed delayed villous maturation, reducing exchange efficiency at the maternal-fetal interface. The ensuing hypoxia and nutrient deficiency trigger a compensatory response of abnormal villous capillary hyperplasia. These capillaries are structurally aberrant, which instead exacerbates placental dysfunction and elevates the risk of adverse pregnancy outcomes, including SGA, fetal hypoxia, and placental abruption (27–29).

Increased syncytial knots are a finding typically observed in placentae with accelerated villous maturation, as syncytial knots are a reflection of placental maturity (18, 30). Our observation of delayed villous maturation in hypothyroidism placentae may therefore explain the lower incidence of syncytial knots in the hypothyroidism group compared to the controls.

Persistent or chronic inflammation of the placenta can result in placental dysfunction, leading to adverse reproductive outcomes (31). In this study, it was observed that compared with the control group, the incidences of CCA and VUE were higher in the hypothyroidism group during pregnancy. Chronic inflammatory lesions of the placenta may be caused by infections such as viruses, bacteria, parasites, or by maternal immune responses to fetal antigens (32). Chronic chorioamniotic inflammation is characterized by infiltration of maternal T-cells from decidua into the chorioamniotic membrane (33). These cytotoxic T-cells induce apoptosis of trophoblast cells, leading to thinning of the chorionic trophoblast layer and damage to the fetal membranes (32). VUE is a destructive villous inflammatory lesion. It’s characterized by the invasion of maternal CD8 + T-lymphocytes into the villous stroma, which can lead to villous sclerosis and necrosis (34). VUE most commonly affects the terminal villi, which are the gas and nutrient exchange sites closest to the maternal surface. CCA is frequently associated with VUE (35).

Currently, only animal studies have shown that hypothyroidism can affect the establishment of an anti-inflammatory environment at the maternal-fetal interface and impact the maternal immune function. The reduced expression of IL-10, NOS2 and IFN-γ in the placenta of rats with hypothyroidism has supported this. In contrast, rats treated with L-thyroxine showed an increase in anti-inflammatory cytokines as well as a decrease in the pro-inflammatory cytokine TNFα in the placenta at mid-gestation (24).

Furthermore, some studies have proposed that in CCA and VUE, placental T - cell chemokines CXCL9, CXCL10 and CXCL11 are overexpressed, and the concentration of CXCL10 in amniotic fluid or maternal and fetal plasma is also significantly increased. The concentration gradient of chemokines promotes the migration of T-cells (36–38). Fallahi et al. found that in patients with hypothyroidism in autoimmune thyroiditis, the circulating levels of CXCL9, CXCL10 and CXCL11 were increased (39). This may also suggest that hypothyroidism is correlated with the occurrence of CCA and VUE.

The observed placental lesions in pregnant women with hypothyroidism, particularly malperfusion-related lesions and inflammatory lesions, reflect placental functional impairment, with potential consequences for offspring health. Studies have shown that MVM-related (e.g., infarcts, retroplacental hemorrhage) and FVM-related (e.g., delayed villous maturation, avascular villi) lesions impair placental maternal-fetal nutrient exchange and vascular perfusion, which are associated with fetal growth restriction, small for gestational age, and even fetal death. It is plausible that the resultant fetal hypoxia and nutrient deprivation could predispose these infants to a higher risk of neurodevelopmental deficits (26, 40, 41). Likewise, VUE and chronic chorioamnionitis are also associated with miscarriage, preterm birth, fetal growth restriction, small for gestational age, and abnormal neurodevelopmental outcome (32, 34, 35). Consequently, these also suggest that future prospective studies on gestational hypothyroidism could incorporate follow-up of the offspring to elucidate its impact on neurodevelopment and assess whether placental pathological alterations can serve as predictive indicators.

No significant differences in the gross morphologic features of the placenta were noted between the two groups. Some studies have described that hypothyroidism can affect the placental weight, placental efficiency, placental thickness or volume to some extent (15, 42, 43). Our results might be related to the fact that our study completely excluded other pregnancy complications and supplementation of levothyroxine. However, additional studies are needed to further analyze this.

The major strengths of our study are the large sample size and the fact that cases with other pregnant complications or infectious diseases were excluded from the study. This might render more accurate evaluation of the impact of hypothyroidism on placental pathological morphology. However, there are some limitations that warrant attention. All the participants in our study were from a single hospital, which may limit the generalizability of our findings. All data were derived from the inpatient records of the final delivery, the exact onset time of hypothyroidism and detailed serial thyroid function tests during the course of pregnancy were not available. Active levothyroxine treatment at delivery serves as a clinical alternative indicator for the management of persistent hypothyroidism during pregnancy, implying that euthyroidism was likely maintained.

## Conclusions

In summary, the findings of our current study indicate that the incidences of placental vascular hypoperfusion and inflammation were higher in hypothyroidism group than that in the control group. This also correlates the pregnancy complications and adverse pregnancy outcomes resulting from hypothyroidism with the corresponding placental function impairment. Based on these findings, future studies could further explore the effects of hypothyroidism on the placenta at different stages of pregnancy, focusing on the underlying molecular mechanisms and clinical significance of the observed histopathological changes, including their associated genetic, immune and endocrine characteristics.

## Data Availability

The original contributions presented in the study are included in the article/supplementary material. Further inquiries can be directed to the corresponding authors.
